# Integrated non-volatile plasmonic switches based on phase-change-materials and their application to plasmonic logic circuits

**DOI:** 10.1038/s41598-021-98418-6

**Published:** 2021-09-22

**Authors:** Rajib Ratan Ghosh, Anuj Dhawan

**Affiliations:** Department of Electrical Engineering, Indian Institute of Technology Delhi, New Delhi, 110016 India

**Keywords:** Nanophotonics and plasmonics, Sub-wavelength optics, Nanophotonics and plasmonics

## Abstract

Integrated photonic devices or circuits that can execute both optical computation and optical data storage are considered as the building blocks for photonic computations beyond the von Neumann architecture. Here, we present non-volatile hybrid electro-optic plasmonic switches as well as novel architectures of non-volatile combinational and sequential logic circuits. The electro-optic switches consist of a plasmonic waveguide having a thin layer of a phase-change-material (PCM). The optical losses in the waveguide are controlled by changing the phase of the PCM from amorphous to crystalline and vice versa. The phase transition process in the PCM can be realized by electrical threshold switching or thermal conduction heating via external electrical heaters or the plasmonic waveguide metal itself as an integrated heater. We have demonstrated that all logic gates, a half adder circuit, as well as sequential circuits can be implemented using the plasmonic switches as the active elements. Moreover, the designs of the plasmonic switches and the logic operations show minimum extinction ratios greater than 20 dB, compact designs, low operating power, and high-speed operations. We combine photonics, plasmonics and electronics on the same platform to design an effective architecture for logic operations.

## Introduction

There is currently a worldwide interest in employing new materials for developing the next-generation data storage and computing systems in order to fulfill the demands of high bandwidth and large information storage^1,2^. Modern computing technology based on von Neumann architecture is reaching its physical limits because of the projected saturation of Moore’s law^3,4^. The drastically increasing trend of bandwidth requirements with less power consumption, new alternative future next-generation computing techniques are required. Photonic computing is supposed to provide a capable alternative to current electronic computing systems due to the uses of optical interconnects in high computing chips instead of electronic interconnects to reduce the power consumptions and to enhance the speeds. Additionally, silicon photonics has emerged as a favorable field due to its capability of effective light modulation, confinement of light and compatibility, with the current complementary metal–oxide–semiconductor (CMOS) fabrication processes^5,6^. The transition from the electronic systems to the photonic systems had started a decade ago. Last few years have seen significant developments in photonic interconnects and circuits due to new material explorations, fabrication technology improvements, and more active research work in this field.

Optical switching and modulations are the key elements in the design of photonic logic circuits. Photonic logic circuits are the main building block behind the optical computations. Several studies in the direction of efficient optical switches, optical modulators, and optical logic circuits have been proposed using the thermo-optic effect, the free carrier dispersion effect, and the pockels electro-optic effect^7–12^. All these devices are volatile in nature i.e., they need continuous voltage to operate, and as a result they have high power consumption. Moreover, they have large interaction lengths due to their weak electro-refractivity. Thus, these devices have large footprints making it challenging to attain the ultra-compact designs using these devices^13–15^.

Over the last few years, a new class of materials called phase change materials (PCMs) have found widespread applicability in both electronics and photonics domain^16–20^. PCMs show changes in electrical and/or optical properties with high-speed switching between two stable states. In recent years, several photonic and plasmonic devices such as on-chip optical modulators and optical switches based on PCMs, have been proposed^21–26^. Ge_2_Sb_2_Te_5_ (GST) is one of the most commonly used PCMs in photonic devices. GST has two self-sustainable phases—the amorphous phase and the crystalline phase—with high contrast in electrical properties and optical properties between the two phases for a broad range of frequencies. It also shows a very low switching time, zero static power, and small footprint—i.e. it can lead to the development of ultra-compact devices. The phase transition of GST can be triggered either electrically (via thermal conduction heating or via joule heating) or optically (self-heating). In the case of electrical triggering, either a voltage is applied across electrodes connected on both sides of a GST film (also called electrical threshold switching or joule heating) or an external heater is used to change the phase of the GST (thermal conduction heating)^27,28^. Moreover, either an on-chip high-intensity laser pulse coupled to the waveguide containing a GST film (on-chip all-optical switching) or a laser beam focused on the GST film (free space all-optical switching) are used to heat up the material in case of optical triggering (also called self-heating)^29,30^. Although any of these heating mechanisms can be used in the electro-optic plasmonic switches being proposed in this paper, we have selected electrical (electrical threshold switching or thermal conduction heating) switching in our design of the plasmonic switches. Since multiple switching elements are present in the plasmonic logic circuits being proposed by us, it is difficult to employ on-chip optical switching due to challenges in routing light. Moreover, multiple laser sources are needed in case of free space optical switching. However, both types of electrical heating mechanisms are suitable for our logic circuits. The advantage of threshold switching in a plasmonic waveguide is that one can apply the electrical signal directly to the metal employed for the plasmonic waveguide. There is no need for extra electrodes or an external heater to apply an external voltage, and that makes the device compact. In our proposed electro-optic switch, the electrical contacts are provided on the metal of the plasmonic slot waveguide coated with a thin layer of GST—i.e. both sides of the GST are connected by the metal electrodes. One can also use an external microheater or the metal in the plasmonic waveguide as a microheater to heat up the GST in case of thermal conduction heating.

Although efficient electronic non-volatile memories and logic circuits based on PCMs have been widely investigated and demonstrated in the last two decades^31–36^, there are only a few reports of PCM-based photonic non-volatile memories and photonic logic circuits^37–41^. There is no previous report describing the design of photonic non-volatile adder circuits or of non-volatile sequential logic circuits. The non-volatile plasmonic switches being proposed in this paper are promising candidates to implement the sequential circuits as memory elements (to store the state of previous outputs) can be designed using these switches. Optical interconnects have a significantly higher bandwidth as compared to electrical interconnects.

In this paper, we first propose a new design of a non-volatile hybrid electro-optic plasmonic switch based on phase change materials in an integrated silicon photonics platform. Thereafter, a microring resonator and an MZI modulator are demonstrated using the plasmonic switch as an active element. Subsequently, we introduce new architectures of non-volatile directed logic using the above-proposed devices, where the non-volatile hybrid electro-optic plasmonic switch is a fundamental unit. After the execution of all the basic logic building blocks, a non-volatile 1-bit half adder circuit is proposed. Finally, non-volatile sequential logic circuits (such as SR, JK, D and T latches) have been presented for photonic integrated circuits. Moreover, to the best of our knowledge, basic logic gates, a half adder circuit, or sequential logic circuits based on the non-volatile plasmonic switches — being proposed in this paper — have not been reported in any previous literature.

### Design of plasmonic switches

**Figure 1:**
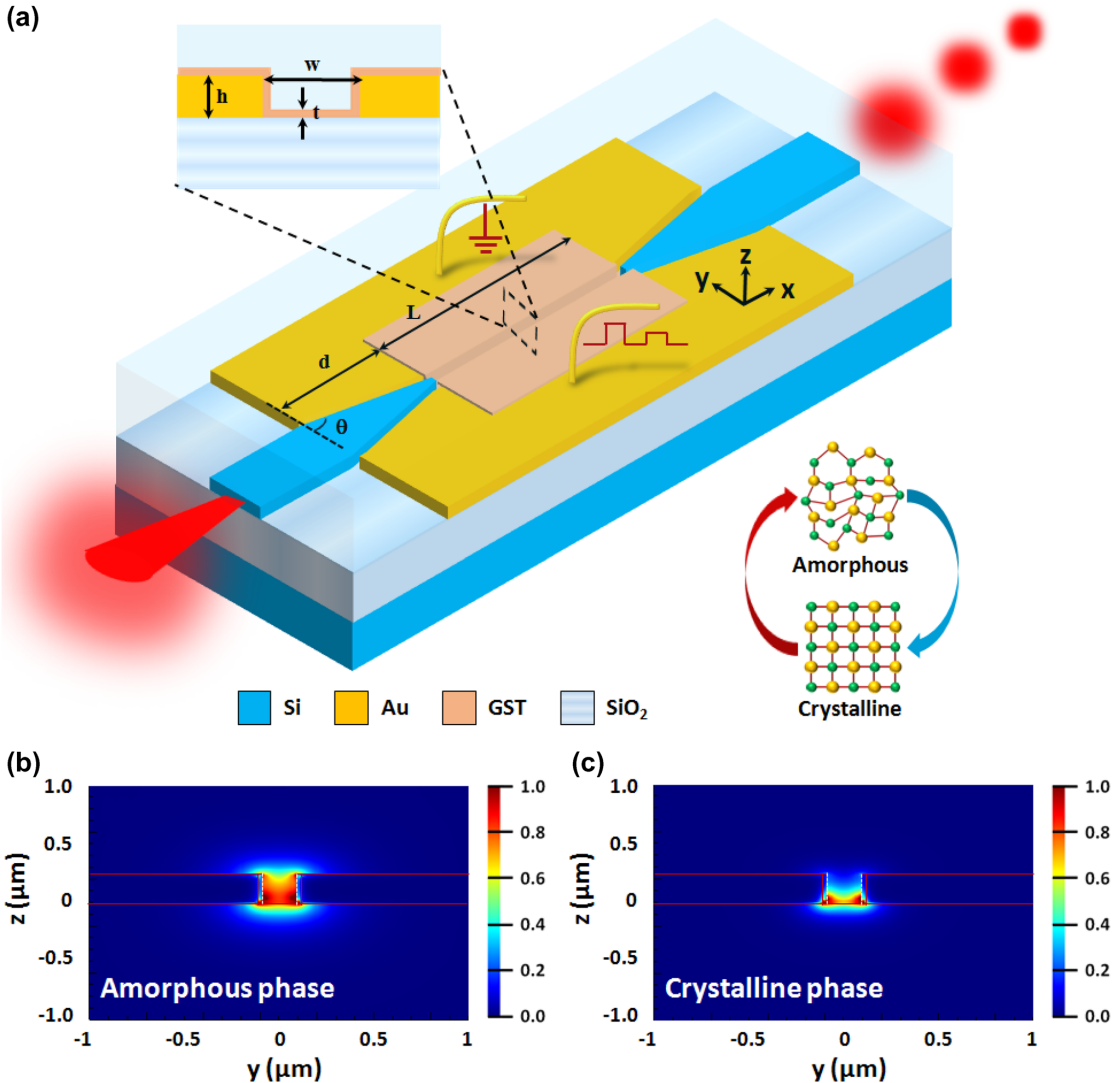
Schematic of the proposed broadband non-volatile hybrid electro-optic plasmonic switch. The inset illustrates the cross-sectional view of the plasmonic slot waveguide with a thin layer of GST. Mode profiles of the PCM (GST) coated plasmonic slot waveguide for the: (**b**) amorphous phase and (**c**) crystalline phase of the PCM at the telecom wavelength of 1550 nm. For both the phases, the mode profiles were calculated for the TE polarization of light. The following optimized geometrical parameters of the PCM coated plasmonic slot waveguide were chosen to calculate the mode profiles—the height of the metal 'h' was taken to be 220 nm, the width of the slot 'w' was taken to be 180 nm, and the thickness of the PCM layer 't' was taken to be 20 nm.

The non-volatile hybrid electro-optic plasmonic switch being proposed in this paper consists of a plasmonic slot waveguide with PCM as an active material coating the surface of the slot waveguide. Figure 1a shows the structure of the proposed plasmonic slot waveguide that uses silicon-gold tapered waveguides, a slot in a gold film to form a plasmonic slot waveguide, and a coating of a PCM such as GST on the surface of the slot waveguide. The structure has three main parts, the first part being a dielectric to plasmonic mode converter in the input section, which is a hybrid silicon-gold tapered waveguide. The second part is a MIM plasmonic slot waveguide coated with a thin layer of GST where the main optical modulation occurs, and the third part is a plasmonic to dielectric mode converter in the output section using a hybrid gold–silicon tapered waveguide. The left top inset of Fig. 1 exhibits the cross-sectional view (in the y–z plane) of the GST-coated plasmonic slot waveguide. The corresponding field distributions of the plasmonic slot waveguide in the amorphous phase and in the crystalline phase of GST are shown in Fig. 1b,c. The input waveguide of the plasmonic modulator is a silicon waveguide on a silica substrate (SOI warfare) with a cross-section of 500 nm × 220 nm. We have chosen the following optimized parameters of the non-volatile hybrid electro-optic plasmonic switch: the height of the Au metal ‘H’ = 180 nm, the slot width of the plasmonic waveguide ‘W’ = 200 nm, the length of the slot ‘L’ = 500 nm, the thickness of the coated PCM material ‘t’ = 20 nm, the length of the taper ‘d’ = 2 µm and the taper angle ‘θ’ = 25º. The top of the device is coated with SiO_2_. The electrical contacts are provided on the metal region of the plasmonic slot waveguide, as shown in Fig. 1. The working mechanism and the different parameter optimizations of the plasmonic switch have been discussed in detail in the supplementary information (see supplementary Sects. 1 and 2).

**Figure 2:**
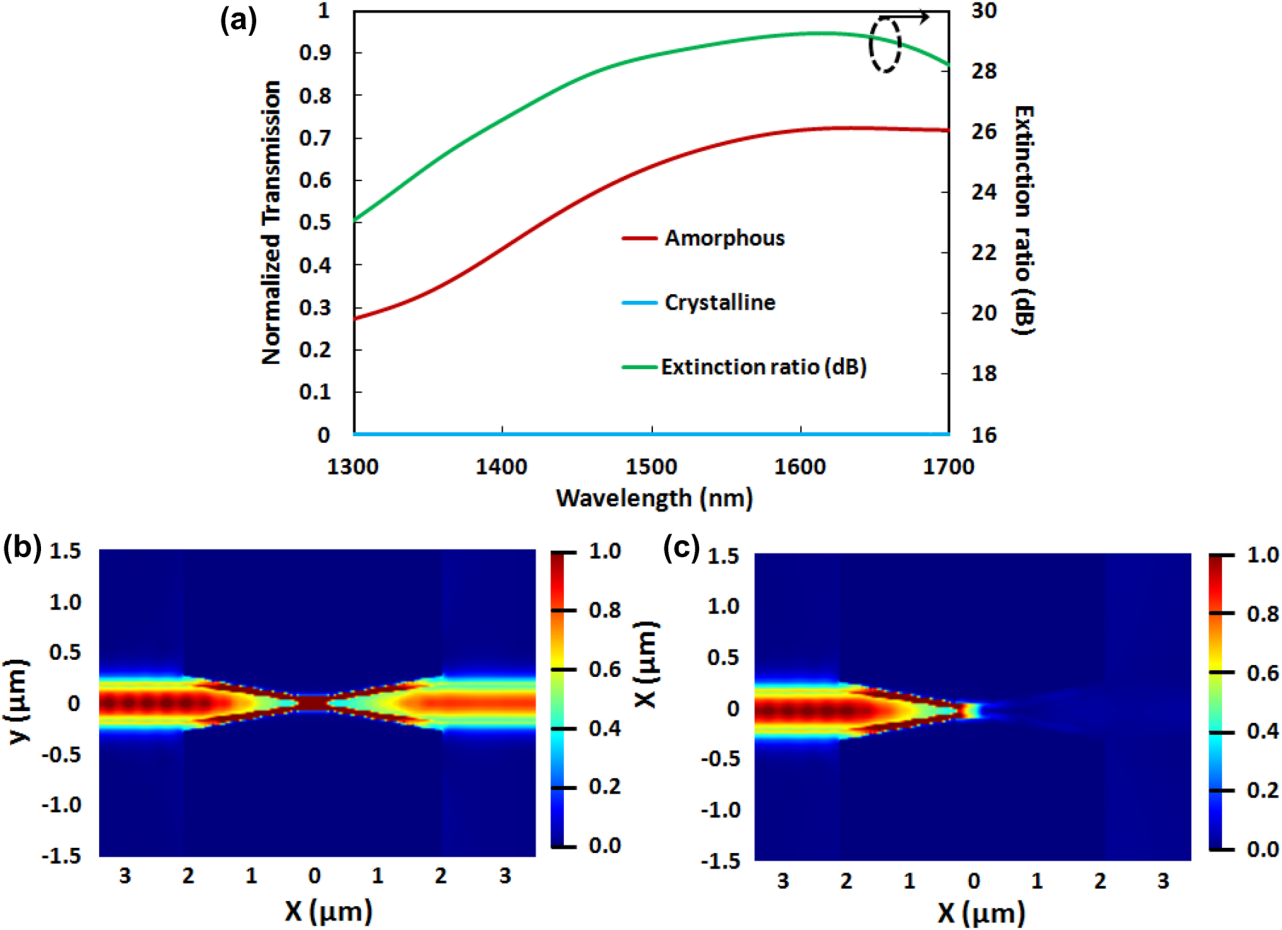
(**a**) The transmission spectra of the non-volatile hybrid electro-optic plasmonic switch for both phases of the GST layer and the extinction ratio of the proposed broadband electro-optic modulator. The optical electric field distributions of the hybrid plasmonic switch at a wavelength of 1550 nm for (**b**) the amorphous phase and (**c**) the crystalline phase. The transmission spectra and field profiles were calculated for TE polarization of light. The following optimized geometrical parameters are chosen for the plasmonic switch—the cross-section of the Si waveguide was taken to be 500 nm × 220 nm, the length of the tapered waveguides was taken to be 2 µm, the taper angle (‘θ’) was taken to be 25°, the height of the metal 'h' was taken to be 220 nm, the width of the slot 'w' was taken to be 180 nm, the thickness of the PCM layer 't' was taken to be 20 nm, and the length of the slot waveguide was taken to be 500 nm.

Figure 2a shows the normalized transmission spectra for both the phases. It can be observed that the optical modes propagate through the slot waveguide due to the high coupling between the hybrid mode of the tapered waveguide and the plasmonic slot waveguide mode (low coupling loss) and low propagation loss in the amorphous phase. The optical field distribution in the amorphous phase is shown in Fig. 2b at 1550 nm. Once the phase of the material is changed to the crystalline phase on applying an external voltage or high-intensity laser pulse, the optical mode hardly propagates through the slot plasmonic waveguide due to the higher coupling loss and the higher absorption loss. Figure 2c shows the optical field distribution in the crystalline phase (OFF state) at 1550 nm wavelength. As a result, the extinction ratio (ER) between the ‘ON’ state and the ‘OFF’ state is very high at 1550 nm. Figure 2a shows the overall extinction ratio spectrum. The extinction ratio is defined as the difference in insertion loss between the ON and the OFF states. The ER spectrum shows the broadband nature of the plasmonic switch with a bandwidth of over 400 nm by considering the operating extinction ratio of > 20 dB. The ER is 29 dB at 1550 nm wavelength. The optimization of the GST thickness of the plasmonic switch is discussed in supplementary Sect. 2 (see Figs. S1(a)–(d)). The Figure-of-Merit (FoM) for a switch to determine its performance is defined as FoM = ER/IL_ON_. The FoM for the electro-optic plasmonic switch being proposed in this paper was calculated to be 21 (at a wavelength of 1550 nm), which is higher than that reported in previous works^27,42–45^. A comparison of our proposed plasmonic switch with other PCM based integrated optical switches—in terms of different switching parameters—is shown in Table S1 (in the supplementary Sect. 4). The effect of the length of the plasmonic slot waveguide on the Insertion loss and Extinction ratio has been shown in supplementary Sect. 2. We have calculated the IL and ER for the waveguide lengths of 250 nm, 375 nm and 500 nm (see Fig. S2 in the supplementary Sect. 2). It shows that the IL reduces with the length of the waveguide due to the metal loss. But at the same time, the ER increases with the waveguide length. Therefore, there is trade-off between IL and ER.

The GST-filled waveguide supports the gap-plasmonic mode and shows a small change in transmission between two phases of the GST in the previously reported work, due to the challenge to electrically switch a significant fraction of GST^42^. The transmission change is very small even after full crystallization. Therefore, the structure has been modified in such a way that it shows a significant change in transmission even with a small volume of GST phase transition. We have taken a Si-Au hybrid plasmonic tapered waveguide on both the sides, a MIM plasmonic slot waveguide and a thin film of GST (thickness of 20 nm) unlike silicon nitride-Au tapered waveguide on both sides, a nanogap in bowtie like structure and totally filled with GST (more than 75 nm thickness). As a result, the structure shows higher transmission changes. A crystalline filament diameter of 25 nm has been considered at the tip of the gap in their simulation results^46^. Since our structure is a MIM plasmonic slot waveguide of length 500 nm, which may create many filaments. The effect of the full crystallization and partially crystallization has been shown in the supplementary information (see supplementary Sect. 6). We have also mentioned few other configurations in terms of phase transition mechanism of the GST film to overcome the above issue (see Fig. S4 of the supplementary Sect. 6). The configurations consist of thermal conduction heating via external heaters (such as graphene heater) and the metallic waveguide as a micro-heater. The heating performance and power calculations have been discussed in detail in the supplementary information (see Fig. S3 of the supplementary Sect. 6)). The switching repeatability and the fabrication process of the plasmonic switch have been discussed in supplementary Sect. 3.

### Microring resonator based EO switch

**Figure 3:**
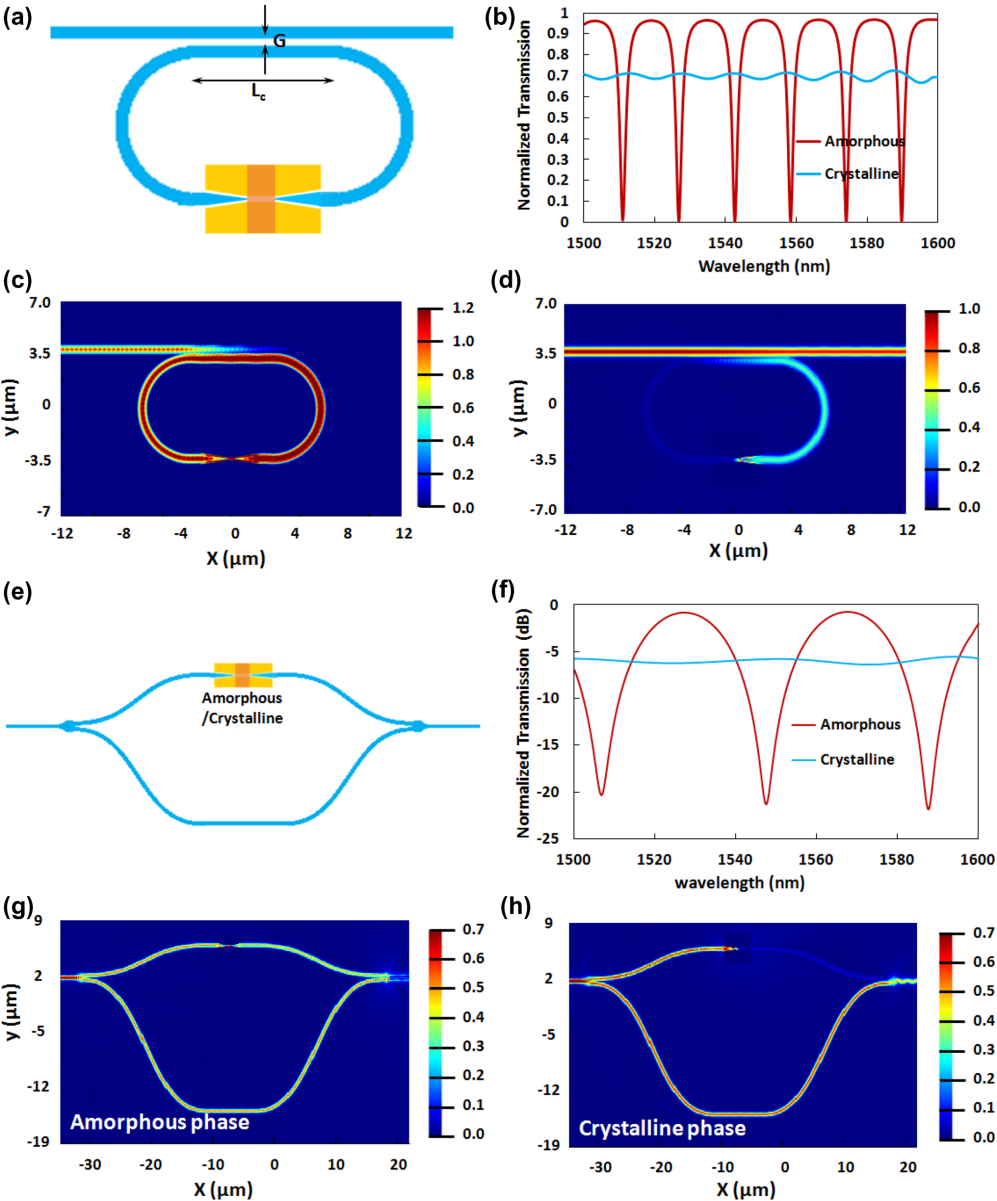
(**a**) Schematic of the racetrack microring resonator based electro-optic switch having the hybrid non-volatile electro-optic plasmonic switch as the fundamental unit. (**b**) The normalized transmission spectra of the racetrack microring resonator based electro-optic switch for the amorphous phase and the crystalline phase of the PCM (GST) layer. (**c**)–(**d**) The optical electric field distribution of the proposed electro-optic switch at the resonance wavelength of 1558 nm for (**c**) the amorphous phase and (**d**) the crystalline phase of the PCM layer. (**e**) Schematic of the asymmetric MZI based electro-optic switch having the hybrid non-volatile electro-optic plasmonic switch as the active element in one arm. (**f**) The normalized transmission spectra of the asymmetric MZI based electro-optic switch for the crystalline phase and the amorphous phase of the PCM (GST) layer in the plasmonic switch. (**g**),(**h**) The optical electric field distribution of the proposed electro-optic switch at the resonance wavelength of 1548 nm for the (**g**) amorphous phase and (**h**) the crystalline phase of the PCM layer.

The proposed EO switch is based on a racetrack microring resonator with the non-volatile hybrid electro-optic plasmonic switch in the circumference of the ring waveguide as an active element (see Fig. 3a). The optical mode is coupled to the ring via a Si waveguide having a cross-section that is the same as that of the Si waveguide of the hybrid plasmonic switch, i.e., 500 nm × 220 nm. The optimized coupling length (L_C_) and a gap (G) between the input waveguide and coupling waveguide of the ring are of 6 µm and 130 nm, respectively. Moreover, the optimal geometrical parameters of the plasmonic switch are the same as the ones described in the previous section (i.e. a metal thickness of 220 nm, plasmonic slot width of 180 nm, and the thickness of the GST layer being 20 nm). Electrodes are connected to the plasmonic switch in a manner shown in Fig. 1 and Fig. S3 of the supplementary Sect. 6. Figure 3b shows the transmission spectra for both phases. When GST is in the amorphous phase, the optical loss in the ring is small. As a result, the optical mode can propagate through the ring resonator. For a proper coupling length and coupling gap between the input waveguide and the ring resonator waveguide, resonance condition is achieved in the ring resonator for certain wavelengths. Light coupled back to the through port of the input waveguide after a roundtrip experiences a 180° phase shift with respect to the light coming from the input port of the input waveguide. Hence, destructive interference occurs between the light coming from the input port and the light coupled back to the through port of the input waveguide. Therefore, there is complete elimination of light exiting the through port at the resonance wavelengths (see Fig. 3c). In contrast, due to the significant optical loss in the crystalline phase, optical mode can’t propagate through the ring waveguide. As a result, no interference happens and the optical power propagates through the through port (see Fig. 3d).

### MZI based non-volatile hybrid plasmonic switch

In this section, we present an asymmetric Mach–Zehnder Interferometer (MZI), as shown in Fig. 3e, based on the hybrid plasmonic switch proposed in this paper. Compact and low loss Y-junction based splitters and combiners have been used to design the asymmetric MZIs^47^. The splitter in the input section splits the input power equally between two arms of the MZI and then the optical powers of the two arms are combined by the combiner in the output section. The hybrid plasmonic switch is embedded in the sorter arm of the asymmetric MZI. The transmission spectra and the field distributions of the MZI for both the phases of GST are shown in Fig. 3f–h, respectively. The details of the working mechanism of the MZI based non-volatile hybrid plasmonic switch are provided in the supplementary information (see supplementary Sect. 5).

### Fundamental non-volatile EO logic operations

**Figure 4:**
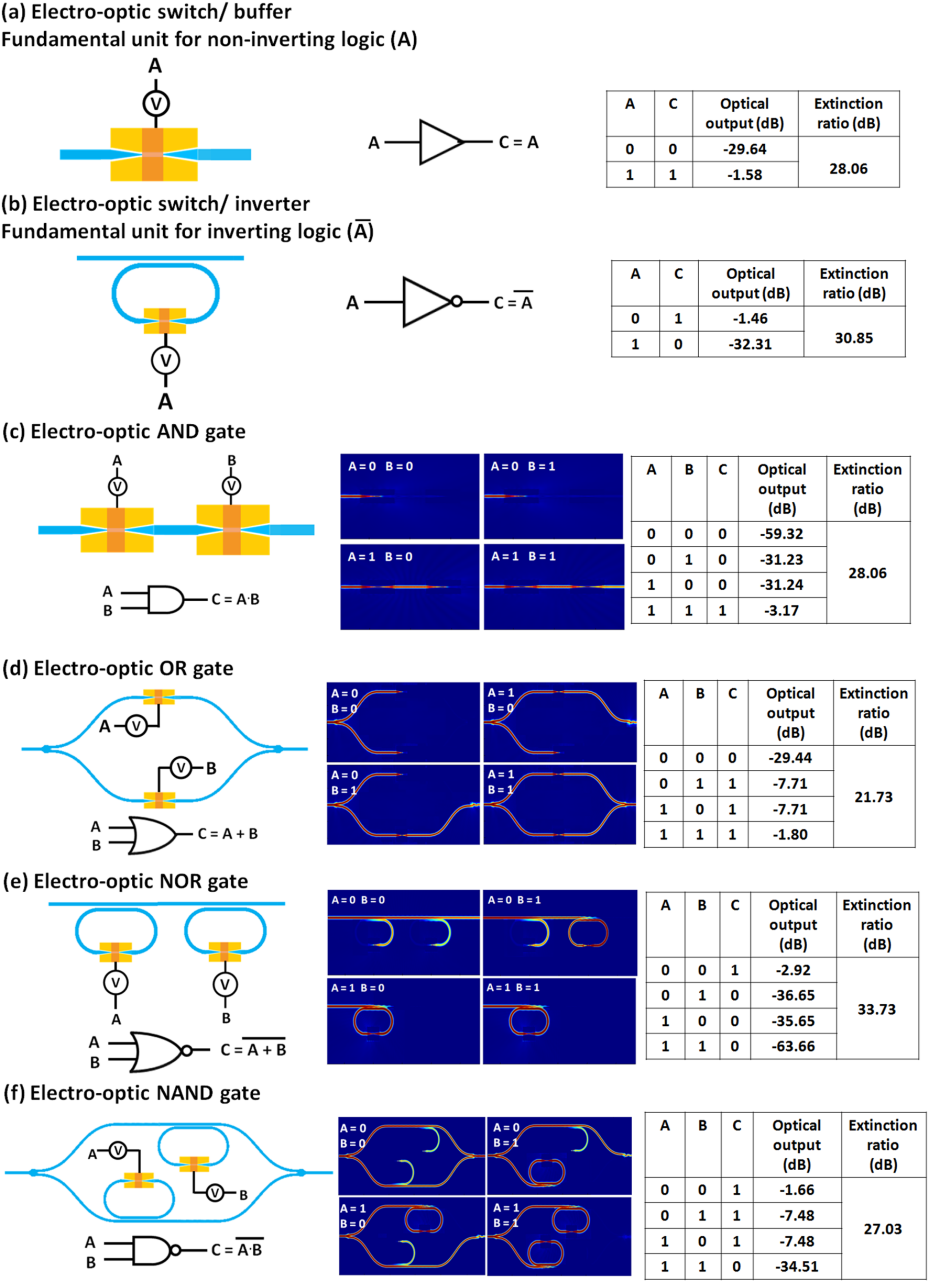
Electro-optic configurations, graphical symbols, and truth tables of: (**a**) fundamental unit of non-inverting logic, (**b**) fundamental unit of inverting logic, (**c**) AND logic, (**d**) OR logic, (**e**) NAND logic, and (**f**) NOR logic.

**Figure 5:**
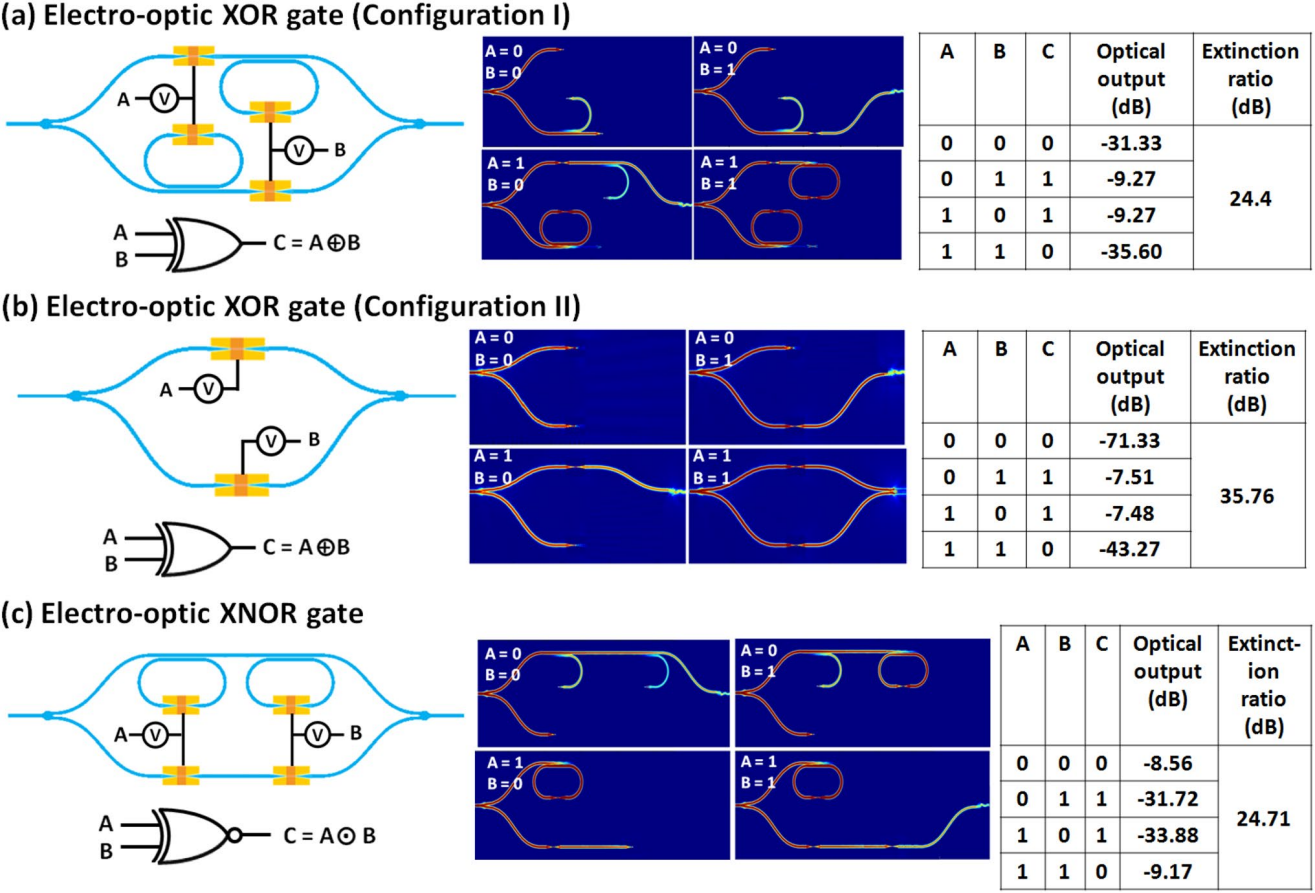
Electro-optic configurations, graphical symbols, and truth tables of: (**a**) XOR logic (configuration 1), (**b**) XOR logic (configuration 2), and (**c**) XNOR logic.

The hybrid non-volatile electro-optic plasmonic switch proposed in this paper is the fundamental switching component for the design of all the combinational logic circuits. This plasmonic switch acts as a fundamental unit for non-inverting logic. Thus, it operates in an ON state in the amorphous phase, and in an OFF state in the crystalline phase. To design logic operations using inverting logic, an electro-optic inverter is needed, such that in the amorphous phase the transmission should be very small (OFF state) and in the crystalline phase the transmission should be high (ON state). This can be done by using the microring resonator-based electro-optic switch proposed above. Thus, it can act as a fundamental unit for inverting logic. Figures 4 and 5 show the schematics of both the fundamental units (non-inverting logic and inverting logic, shown in Fig. 4a,b, as well as all six basic logic gates (i.e., AND, OR, NAND, NOR, XOR, and XNOR) containing the proposed hybrid non-volatile electro-optic plasmonic switch as an active element. Figure 4c shows the architecture of an electro-optic AND gate, which can be designed by using two plasmonic switches. Two non-inverting fundamental logic units are connected in series in this architecture, which defines the logical multiplication (i.e. the ‘AND’ operation). The electrical inputs (A and B) are connected to the plasmonic switches. When one input (or both the inputs) is in logic ‘0’, the corresponding transmissions in the output port are low due to the high loss in the crystalline phase, as shown in the truth table and field profiles. When both the inputs are logic ‘1’, which means both the switches are in the amorphous phase, the transmission in the output port is high. The corresponding truth table and electrical schematic are also shown in Fig. 4c. The extinction ratio was calculated in the output port between the highest transmission in OFF state and lowest transmission in ON state. The calculated extinction ratio for this electro-optic AND gate architecture is 28.06 dB. The optical field profiles for different input combinations are shown in Fig. 4c. However, logical addition (i.e. the ‘OR’ operation) can be implemented by connecting two EO switches in parallel using the splitter in the input section and combiner in the output section. In the case of OR logic gate, two non-inverting fundamental logic units are cascaded in parallel, as shown in Fig. 4d. When one input is logic ‘1’ (or when both the inputs are logic ‘1’), the corresponding arm passes the optical power. Thus, the transmissions in two arms are getting added using the combiner and the output shows high transmission. On the other hand, when both outputs are in logic ‘0’, transmission in output port is very low. The calculated extinction ratio is 21.73. In this case, the insertion losses in ‘ON’ state are high compared to the AND gate due to the splitting of optical power between the two arms. The NOR and NAND gates require two inverting fundamental logic units — connected in series and parallel, respectively, as shown in Fig. 4e and f. All the above-discussed logic gates need two EO switches. However, in the case of XOR and XNOR logic implementations, four EO switches are required for each logic implementation (see Fig. 5a,c). However, the XOR logic gates can also be implemented with two EO switch based on an asymmetric MZI having the hybrid non-volatile electro-optic plasmonic switch in both arms of the MZI (see Fig. 5b). The difference of the arms-lengths is such that the phase difference between the two arms is 180° in the amorphous phase. The difference between arm lengths is 5.8 µm. When both the inputs are logic ‘1’ (i.e. both the non-inverting electro-optic plasmonic switches are in the amorphous phase), light passes through both the arms with a phase difference of 180°. Thus, the transmissions of both the arms destructively interfere in the combiner and the net transmission in the output port is low. In contrast, when any one input is logic ‘0’, the output shows the high transmission as there is transmission through only one of the arms of the MZI. The transmission is low in case of logic ‘0’ of both inputs as there is no transmission through any of the arms the MZI. The extinction ratio is high and the insertion loss in ON state (IL_ON_) is low as compared to the configuration 1 of the XOR gate. The optical field distribution profiles of all the logic gates for the different combinations of logic inputs are shown in the corresponding figures (see Figs. 4 and 5). Moreover, any combinational logic circuit can be implemented using these basic logic gates (AND, OR, NAND, NOR, XOR, XNOR), which in turn are implemented using the two fundamental units (i.e. inverting logic units and non-inverting logic units). Table S3 in the supplementary information (see supplementary Sect. 7) provides a comparison of the performance of our proposed plasmonic logic gates and circuits with that of the different previously reported optical logic gates.

### Half Adder circuit

**Figure 6:**
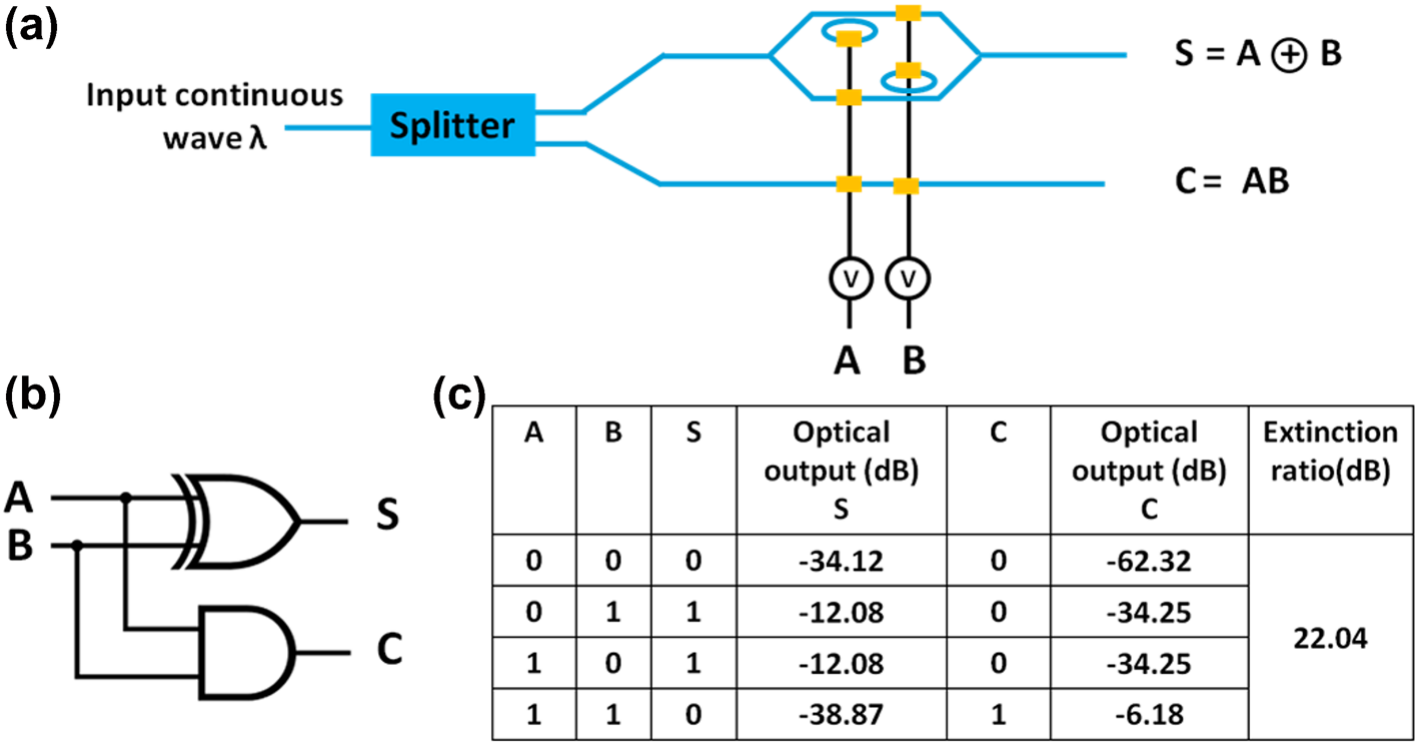
(**a**) Electro-optic architecture, (**b**) graphical symbol, and (**c**) truth table of a 1-bit half adder circuit. While the blue colored connecting lines indicate the optical connections using silicon waveguides, the black colored connecting lines indicate the electrical connections using copper transmission lines.

Figure 6a shows the architecture of a 1-bit half adder non-volatile electro-optic combinational logic circuit. It can add two input binary bits employing a digital circuit shown in Fig. 6b. The outputs of a half adder circuit (i.e., sum S and carry C) in terms of inputs (i.e., input bits A and B) are:

$$\begin{aligned} & {\text{S}} = {\text{A}} \oplus {\text{B}} \\ & {\text{C}} = {\text{AB}} \\ \end{aligned}$$

The half adder circuit consists of two splitters, one combiner, four non-inverting logic units and two inverting logic units. The S-parameters of the plasmonic electro-optic switches (described in the section titled ‘Design of plasmonic switches’) and optical waveguides were imported to an S-Parameter Simulator (SPS) solver (Lumerical Interconnect) to calculate the transmission for different combination of inputs. The solver calculates frequency domain response (such as amplitude, phase, group delay and dispersion) of a photonic integrated circuit by representing the different circuit elements as scattering matrices. The truth table of the 1-bit electro-optic half adder circuit is shown in Fig. 6c. All the logic implementations are based on the electro-optic switching. The extinction ratio of the proposed half adder circuit is 22 dB.

### Sequential non-volatile asynchronous EO logic (Latch) circuits

**Figure 7:**
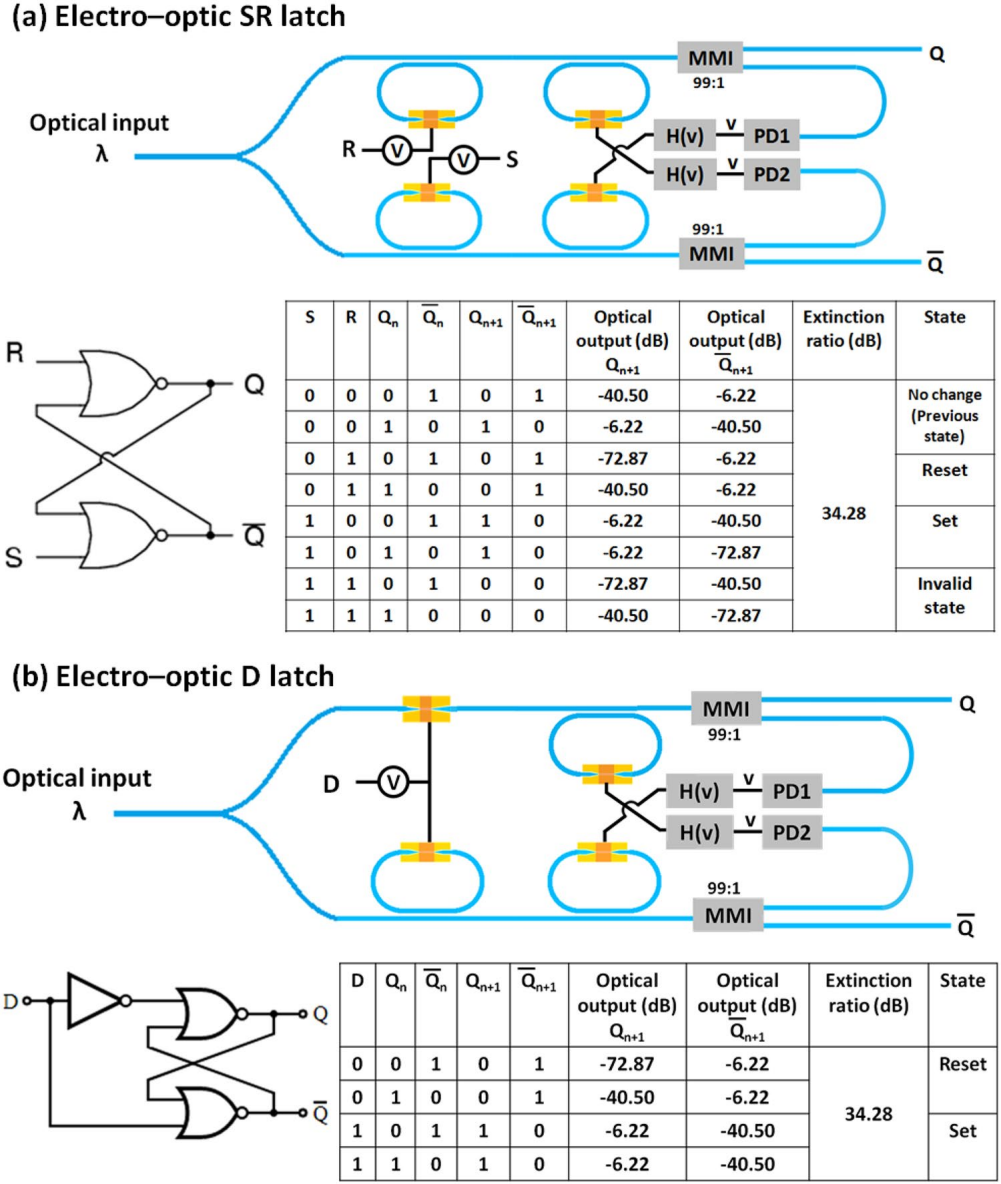
Electro-optic configurations, graphical symbol, and truth table of a: (**a**) SR latch circuit, and (**b**) D latch circuit. Where, PD and H(v) represents the photodetector and conditional electronic circuits respectively. H(v) generates necessary voltage pulse with different amplitude and duration for amorphorization and crystallization depending on the photodetector output.

**Figure 8:**
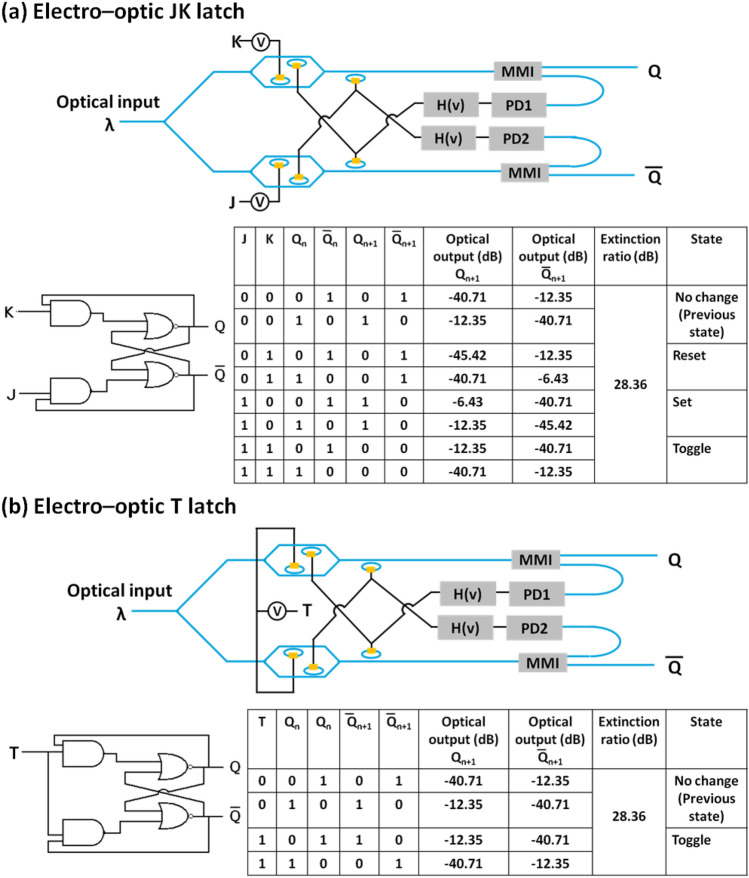
Electro-optic configurations, graphical symbol, and truth table of a: (**a**) JK latch circuit, and (**b**) T latch circuit.

In contrast to combinational logic circuits (whose outputs only depend on the present inputs), outputs of a sequential logic circuit depend not only on the present input but on the sequence of past inputs. Thus, in the case of sequential logic circuits, feedback paths are needed. Both combinational and sequential logic circuits have significance to design practical digital circuits. In general, the sequential logic circuits are needed to implement the finite state machines, which are basic building block in digital circuitry. Sequential digital circuits are two types — synchronous and asynchronous. The outputs of the synchronous sequential circuits change in the response of the clock signal. In contrast to that, the outputs can change at any time in the response of the inputs in case of asynchronous sequential circuits. In this section, non-volatile EO asynchronous sequential logic circuits have been designed. We have demonstrated all four types of asynchronous latch circuits i.e., a SR latch, a D latch, a JK latch and a T latch (see Figs. 7 and 8). To design the sequential logic circuits, photodetectors (PDs) and conditional electronic circuits (H(v)) have been used. The conditional circuit generates the voltage pulse (H(v)) according to the output voltage (v) of the photodetector, which in turn depends upon the optical intensity. The photodetector converts the optical signal to an electrical signal. The electrical signals are used to trigger the conditional electronic circuit, which generates two types of electrical pulse signals (with the two types having different amplitudes and pulse width) to change the phase of the GST from amorphous phase to crystalline phase and vice versa (i.e., for crystallization and amorphization) in the plasmonic switch. The NOR gate based SR latch circuit is shown in Fig. 7a. A continuous optical input signal has been used. The input optical power is divided into two arms using a splitter, and EO NOR gates are implemented in both the arms. While the ‘S’ input is connected to the first input of the NOR gate implemented in the upper arm, the ‘R’ input is connected to the first input of the NOR gate implemented in the second arm. Moreover, the second input of the NOR gates implemented in both the arms are connected to the two conditional circuits shown in Fig. 7a. The outputs of the NOR gates first enter a splitter which routes a portion (~ 1%) of the optical power to a high-speed on-chip photodetector. The splitter can be a directional coupler. The photodetector converts the optical intensity into a voltage signal. Now, the voltage signal triggers the conditional electronic circuit, which generates the voltage pulse according to the voltage signal output of the photodetector. This process acts as a feedback path. When both inputs (S and R) are in logic ‘0’, optical mode passes through the first inverting EO switch and then passes through the second EO switch depending on the previous outputs. If the $Q_{n}$ is in logic ‘0’ (i.e., $\overline{Q}_{n}$ = 1), the electrical signal in the photodetector (PD2) output is high and the conditional electronic circuit generates a voltage pulse for amorphization. Thus, optical modes cannot propagate through the upper arm and the output of the NOR gate $Q_{n + 1}$ is logic ‘0’. Due to the low intensity, the electrical signal in the photodetector (PD1) output is low. Thus, the conditional electronic circuit generates a voltage pulse for crystallization. This changes the phase of the GST in the lower arm EO switch. As a result, optical mode passes through the EO switch and the output of the NOR gate $\overline{Q}_{n + 1}$ is logic ‘1’. Thus, the state of the SR latch is the same as the previous one for S = 0 and R = 0, which is called as the ‘No change state’. When the inputs S = 0 and R = 1, the optical mode cannot pass through the first EO switch in the upper arm. Thus, the output of the NOR gate $Q_{n + 1}$ is logic ‘0’ irrespective of the logic state of the $\overline{Q}_{n}$. The photodetector output (PD1) is low and the conditional circuit generates the electric pulse for crystallization. As a result, the optical mode can propagate through both the EO switches in the lower arm and the output of the NOR gate $\overline{Q}_{n + 1}$ is logic ‘1’. This state is called the ‘Reset state’. Similarly, for inputs S = 1 and R = 0, the outputs $Q_{n + 1}$ = 1 and $\overline{Q}_{n + 1}$ = 0 (Set state). When both the inputs are logic 1, both outputs are in logic ‘0’. This state is invalid. The corresponding truth table and the electrical schematic are also shown in Fig. 7a. The extinction ratio was calculated in the output ports between highest transmission in the OFF state and lowest transmission in the ON state. The calculated extinction ratio is 34.28 dB for this EO SR latch. In this work, we have calculated the steady-state frequency domain analysis. The transient response and timing analysis are beyond this paper. The working mechanism and output for different input combinations of the different state of the truth table for D latch (see Fig. 7b), JK latch (see Fig. 8a), and T latch (see Fig. 8b) have been discussed in detail in the supplementary information (see supplementary Sect. 8).

## Conclusions

In summary, we have proposed and modelled a novel design of a non-volatile broadband hybrid EO plasmonic switch based on an electrically controlled phase change material ‘GST’ embedded in a plasmonic slot waveguide. The proposed switch exhibits excellent performance in several important categories, including large extinction ratio (> 28 dB), high bandwidth (BW > 400 nm), low power consumption, and low footprint (length of the GST is 0.5 µm). The structures proposed in this paper are easy to fabricate and are compatible with non-volatile photonic integrated circuits and silicon photonics. Using this hybrid plasmonic switch as an active element, other EO switches based on ring resonators and MZIs have also been proposed. It was demonstrated that basic logic gates (i.e. NOT, AND, OR, NAND, NOR, XOR and XNOR), a plasmonic combinational logic circuit such as a 1-bit half adder circuit, as well as plasmonic asynchronous sequential latch circuits (i.e., SR, JK, D, and T latch) can be implemented using the plasmonic switches as the active elements. These devices can be useful in the fields of optical computing, optical memories, in-memory computation, and signal processing.

## Methods

We have used a 2D Finite Difference Eigenmode (FDE) solver (Lumerical MODE) to calculate the effective refractive indices, spatial field profiles, and the optical losses in the plasmonic slot waveguides at a wavelength of 1550 nm employing a mesh size of 2 nm. The FDE solver calculates the frequency dependence of modes, spatial mode field profiles, the effective refractive indices, and optical losses in waveguides by meshing the cross-sections of the waveguides (using the finite-difference algorithm) for solving the Maxwell's equations.

Three-dimensional Finite Difference Time Domain simulations (using Lumerical FDTD) of the proposed structure were also carried out to verify the performance in terms of optical transmission. We have taken a mesh size fine enough to ensure the results remain almost same even after reduction of the mesh size. We have employed perfectly matched layer (PML) boundary conditions in all three directions in our simulations.

For all the optical simulations, the Palik model has been used to model the refractive indices of the different materials such as Si, SiO_2_ and Au employed in this paper. As the experimental work by the Majumdar et al. has provided the complex refractive indices of the amorphous and the crystalline phases of GST^48^, we have employed these refractive indices of GST in this paper.

## Supplementary Information

Supplementary Information.
