# Supplementary material for: Integrated non-volatile plasmonic switches based on phase-change-materials and their application to plasmonic logic circuits
Source: Sci Rep. 2021 Sep 22;11:18811. doi: 10.1038/s41598-021-98418-6 (PMC8458359; doi:10.1038/s41598-021-98418-6)
Supplement: Supplementary file 1 — Supplementary Information. [file 41598_2021_98418_MOESM1_ESM.docx]

SUPPLEMENTARY INFORMATION

for

**Integrated non-volatile plasmonic switches based on phase-change-materials and their application to plasmonic logic circuits**

# Rajib Ratan Ghosh1, and Anuj Dhawan1, *

1 Department of Electrical Engineering, Indian Institute of Technology Delhi, New Delhi, 110016, India

* [adhawan@ee.iitd.ac.in](mailto:corresponding.author@email.example)

# 1. Working principle of the plasmonic switch

The proposed non-volatile hybrid electro-optic plasmonic switch works on the following process: (1) the dielectric mode from the input silicon waveguide is coupled to the plasmonic mode in the slot waveguide through the silicon-gold tapered waveguide region. The coupling efficiency is based on the refractive index of the PCM due to the index matching condition between the tapered waveguide and plasmonic slot waveguide. (2) The plasmonic mode propagates through the slot waveguide with low absorption loss in the amorphous phase and high absorption loss in the crystalline phase. (3) The plasmonic mode from the slot waveguide is coupled to the dielectric mode in the output silicon waveguide through the silicon-gold tapered waveguide region. Two key parameters of the integrated electro-optic switches are low insertion loss in the ‘ON’ state (IL_ON_) and a high extinction ratio (ER). These two parameters are directly related to the complex refractive indices of both the phases of the GST in our proposed hybrid electro-optic plasmonic switch ⎯ as the complex effective refractive index of the plasmonic slot waveguide (which is responsible for the modulation of the optical power) depends on the complex refractive index of the GST. To reduce the insertion loss in the ‘ON’ state of the hybrid plasmonic switch, two conditions have to be followed. Firstly, the extinction coefficient of the PCM in the amorphous state (ON state) should be as small as possible, as a result of low absorption loss in the slot waveguide. Secondly, the real part of the effective refractive index of the PCM coated plasmonic slot waveguide in the amorphous state should be such that it shows an effective coupling between the input waveguide and the slot waveguide by index matching between the hybrid taper mode and the plasmonic slot waveguide mode, as a result of low coupling loss. Moreover, the complex refractive index difference between the two phases of the GST is comparatively high, as a result of high coupling loss in the crystalline phase. Due to these reasons, the extinction ratio of the proposed hybrid electro-optic plasmonic switch is very high.

# 2. Optimization of different parameters of the plasmonic switch

The objective of the optimization process of the proposed hybrid electro-optic plasmonic switch is to increase the extinction ratio and reduce the insertion loss (IL_ON_) in ‘ON’ state. This is achieved by the minimization of the optical loss in the amorphous phase (‘ON state’) and by the maximization of the optical loss in the crystalline phase (‘OFF state’). The insertion loss is the combined loss comprising of the coupling loss from the hybrid mode in input tapered waveguide to the plasmonic slot waveguide mode, the coupling loss from the plasmonic slot waveguide mode to the hybrid mode in output tapered waveguide, and the absorption losses in the metal and the GST. The absorption loss due to the metal is the same for both phases. In contrast, the absorption loss in the PCM (GST) and the coupling loss are different for both phases of the PCM. The optical loss reduces with higher thicknesses of the metal and also with higher slot widths. Thus, the optimized metal thickness of 220 nm and a plasmonic slot width of 180 nm have been taken. Figure S1 (a) shows the variation of the real part of the effective refractive index of both the phases, as well as that of the change in the real part of effective refractive index variation of the slot waveguide, with the thickness of the GST film. The variation of the optical loss in the plasmonic slot waveguide for both the phases, as well as that of the extinction ratio in the slot waveguide, as a function of GST thickness is shown in Fig. S1(b). Figure S1(d) shows the effective complex refractive index and the corresponding field profiles at different positions of the input silicon-gold tapered waveguide (see Fig. S1(c)). The effective refractive index of the position 4 and the position 5 (in amorphous phase) should have the approximately same value for effective coupling of light. The thickness of the PCM played


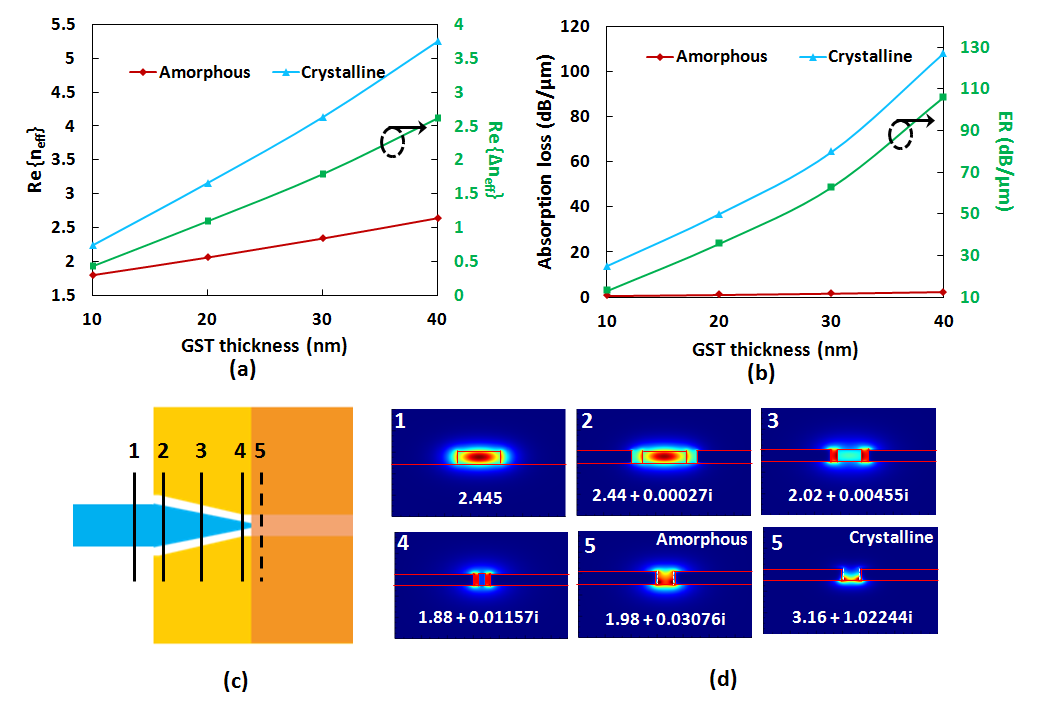


**Figure S1.** (a) Real part of effective refractive index (n_eff_) for both phases of GST and change in the effective refractive index of plasmonic slot waveguide as a function of PCM (GST) layer thickness. (b) Variations in absorption losses for both phases of GST and the corresponding extinction ratio of the slot waveguide as a function of PCM layer thickness. (c) Schematic of the input section of the proposed non-volatile hybrid electro-optic plasmonic switch. (d) Corresponding field profiles at different positions, marked in the schematic of the input taper waveguide. All the fields were calculated at the wavelength of 1550 nm and for TE polarization of light. The following optimized geometrical parameters of the PCM coated plasmonic slot waveguide were chosen ⎯ the height of the metal 'h' was taken to be 220 nm, the width of the slot 'w' was taken to be 180 nm, and the thickness of the PCM layer 't' was taken to be 20 nm.

a very important role in index matching. We have taken the PCM thickness such that the effective refractive index of the PCM coated plasmonic slot waveguide matches with the effective refractive index of the position 4 of the tapered waveguide. We have taken the PCM thickness to be 20 nm to achieve a high extinction ratio (ER) with a relatively low insertion loss (IL_ON_) using a smaller plasmonic slot waveguide length. The coupling loss is very less as the effective refractive index at positions 4 and 5 are approximately the same in the amorphous phase. Moreover, a significant effective refractive difference between these two positions for GST film thickness of 20 nm is responsible for high coupling loss in the crystalline phase. As a result, high extinction ratio and low insertion loss have been achieved with a smaller active region compared to the other previous reported works^1,2^. Moreover, the absorption loss depends on the length of the plasmonic slot waveguide. There is a trade-off between IL_ON_ and the extinction ratio. Thus, to reduce the insertion loss in the ON state with a comparatively high extinction ratio, we have chosen the slot length of 500 nm. We have also calculated the IL_ON_ and corresponding ER for the different lengths of the slot waveguide i.e., 250 nm, 375nm and 500 nm (see Fig. S2).


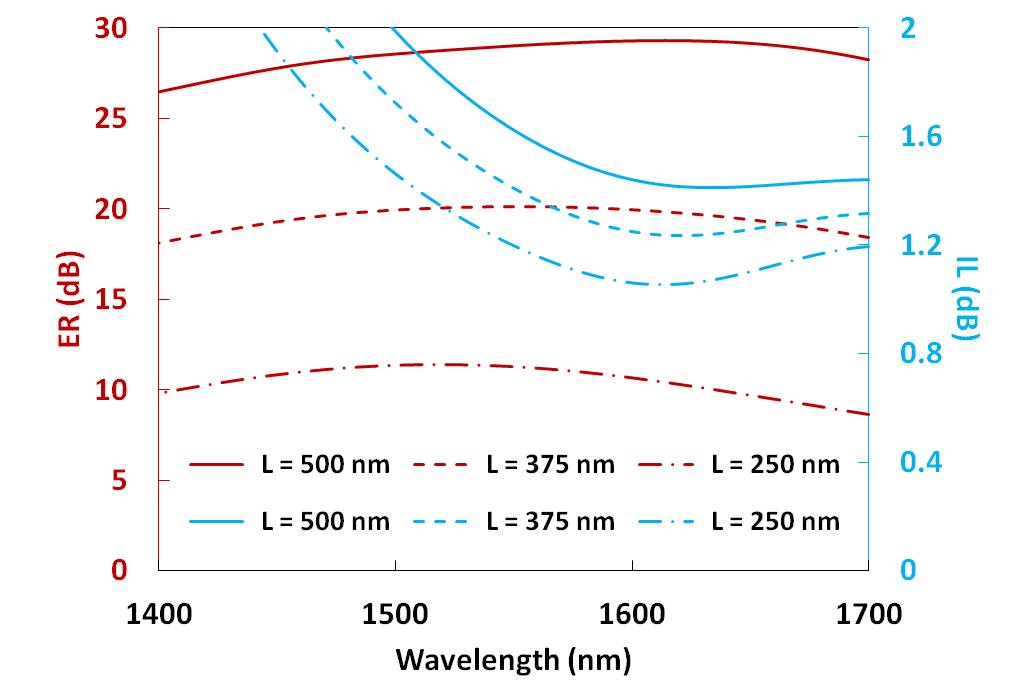


**Figure S2.** The extinction ratio and insertion loss for the different slot length of the plasmonic switch.

# 3. Discussion on repeatability and fabrication of the plasmonic switch

Our plasmonic switch is based on a plasmonic slot waveguide with a thin layer of a phase change material (a 20 nm layer of GST) coating the slot waveguide region ⎯ where the reversible phase transitions can be triggered by directly passing an electrical current through GST (Joule heating). The endurance of the phase change material (GST) has been demonstrated to be very high ⎯ with more than 10^12^ switching cycles between the amorphous and crystalline states, a long retention time (~ 10 years), and an impressive yield characteristics for memory applications^3-6^. Hence, the plasmonic switch described in this paper can be repeatedly switched between the amorphous and crystalline states.

Moreover, the plasmonic switches described in this paper can be easily and repeatedly fabricated using a process that is compatible with the current CMOS fabrication technology. The silicon input and output waveguides can be fabricated by employing electron beam lithography (EBL) followed by reactive ion etching (RIE) of a 220 nm thick silicon layer in a SOI wafer. Subsequently, a second EBL step can be employed to develop windows, through which a 220 nm thick layer of gold can be deposited using electron beam evaporation or thermal evaporation. This can be followed by a gold lift-off step. In order to deposit the GST, a rectangular opening can be first formed using a third EBL writing process on the plasmonic slot waveguide region. A 20 nm GST can be sputtered, followed by liftoff of the GST layer in hot acetone employing sonication^7^, and finally by deposition of a SiO_2_ cladding layer. Hence, our proposed plasmonic switch structure is repeatable from the switching point of view with high endurance and long retention time and from the fabrication point of view due to its compatibility with the CMOS fabrication process.

# 4. Comparison of our proposed plasmonic switch with other PCM based integrated optical switches

A comparison of our proposed plasmonic switch with other PCM based integrated optical switches ⎯ in terms of different switching parameters ⎯ is shown in Table S1 below:

Table S1. Comparison of switching performance for different device types and PCMs

| Device Type | Active material | Modulation mechanism | IL_ON_ (dB/µm) | ER (dB/µm) | FOM | Switching mechanism | BW  (nm) | Length of the active material (µm) |
| --- | --- | --- | --- | --- | --- | --- | --- | --- |
| Si/VO_2_/Au plasmonic modulator [8] | VO_2_ | Non-resonant | 12.5 | 8.9 | 0.71 | Electrical | > 100 | 0.56 |
| Si/ITO/GST waveguide switch [9] | GST | Non-resonant | 0.73 | 10.13 | 13.87 | Electrical | > 100 | 3.0 |
| Si/GST microring [10] | GST | Resonant | 2.5 [dB] | 12 [dB] | 4.8 | Optical | < 0.2 | 3.0 |
| SiNx/GST  MZI switch [11] | GST | Resonant | 7 [dB] | 11 [dB] | 1.57 | Electrical/  optical | < 10 | 5.0 |
| Plasmonic waveguide switch  (Our design) | GST | Non-resonant | 1.71 | 36 | 21 | Electrical/Optical | > 100 | 0.5 |

# 5. MZI based on non-volatile hybrid plasmonic switch

The asymmetric Mach–Zehnder Interferometer (MZI), shown in Fig. 3(e) of the main paper, consists of compact and low loss Y-junction based splitters and combiners, while the hybrid plasmonic switch is embedded in the sorter arm of the asymmetric MZI. The Mach-Zehnder interferometer has been designed with a difference between the arms-length (ΔL) of 24.2 µm. The proposed non-volatile asymmetric MZI is ultra-compact compared to the other MZI structures^12-14^. The resonant wavelength (λ_r_) at m_th_ order mode depends on the difference between the arm length (ΔL) and also the effective refractive index difference (∆n_eff_) of the MZI as given by Eq. (1).

$$m\cdot\lambda_{r}=n_{eff}\left( \Delta L \right)+ (\Delta n_{eff})L \ldots\ldots\ldots\ldots\ldots.(1)$$

The transmission spectra of the MZI for both the amorphous and the crystalline phases of the GST are shown in Fig. 3(f) of the main paper. The MZI is designed for operation with the transverse electric (TE) polarization of light. When the GST film is in the amorphous phase, the optical modes propagate through both the arms and combine in the output combiner constructively or destructively according to the corresponding phase differences. One can observe low transmission for particular frequencies where the phase difference is 180° (i.e., destructive interference). The field distribution of the asymmetric MZI is shown in Fig. 3(g) of the main paper for the amorphous phase. The optical powers are not equal in both the arms (due to the presence of the losses in the plasmonic switch in one arm). As a result, the interference is not proper due to different optical power in the two arms. When the phase of the GST changes from amorphous to crystalline on applying an external voltage to the plasmonic switch, the optical mode hardly propagates through the upper arm of the MZI (i.e., containing the plasmonic switch). As a result, there is no interference in the output combiner, and high transmission is seen in the spectrum for the crystalline phase of the GST film (see Fig. 3(h) of the main paper). But the insertion loss in ‘ON’ state is high due to the splitting of the optical power in the input side. Thus, an optical switch is demonstrated with the inverse operation (i.e., high transmission in the crystalline phase and low transmission in the amorphous phase at resonance wavelength) by switching the phase of the GST.

# 6. Heating performance of the plasmonic switch

Usually, two types of self-heating mechanisms are used to change the phase of the PCM materials, i.e., photothermal heating and joule heating. The photothermal heating mechanisms have been used for all-optical applications, which involve a high-intensity free space laser pulse focused onto the PCM or a high-intensity laser pulse coupled to the PCM layer using an optical waveguide. PCM absorbs the light intensity and therefore gets heated up to the desired temperature. On the other hand, joule heating is used in the case of electro-optic applications, wherein the PCM is conducting. In the case of joule heating, two electrodes are employed across which an external voltage is applied, as shown schematically in Figure S3 (c). Due to some disadvantages of photothermal heating particularly in our plasmonic switch structure, such as nonlinear heating, light routing, as well as alignment issues, we have used the joule heating mechanism (electrical threshold switching) as the mechanism of changing the phase of the PCM in our design of the plasmonic switches. For our device, electrical threshold switching is an effective mechanism due to the advantages such as small applied voltage, and effective light-matter interaction. Two other phase change mechanisms based on electrical conduction heating can also be employed (see Fig. S4) for achieving high levels of crystallization upon heating. We have employed a time-dependent electro-thermal model based on the Finite Element Method (using Lumerical DEVICE), to analyze the heating performance in the process of phase transition. The solver calculates the thermal response using the coupled system of equations for heat transport and conductive electrical transport in an electrically driven Joule heating system. The thermoelectric properties of the different materials used in our simulations are summarized in Table S2. We initially carried out the simulations to study the process of crystallization. When a 8 V pulse is applied for 65 ns, the applied voltage is enough to raise the temperature of the GST film above its crystallization temperature (413 K), but the temperature remains below the melting temperature (823 K) of GST as shown in Fig. S3(a). The energy consumption for the phase transition from amorphous phase to crystalline phase is 358.4 pJ. However, in the process of amorphization, a pulse of 18 V was applied for 10 ns to heat up the GST film above the melting temperature. During the amorphization, the energy consumption is 116.8 pJ. Therefore, the total energy consumption per cycle is 475.2 pJ. The energy is consumed only during the phase transition and it can be reduced by using multi-level operations^15^. Fig. S3(b) shows the transient response of the temperature and heat profile of the above-mentioned amorphization processes. One can observe from the temperature distribution profiles that the heat is generated mainly in the bottom of the slot waveguide. Therefore, the phase transition (from amorphous to crystalline) happened only in the bottom portion of the GST film and other portions remain in the


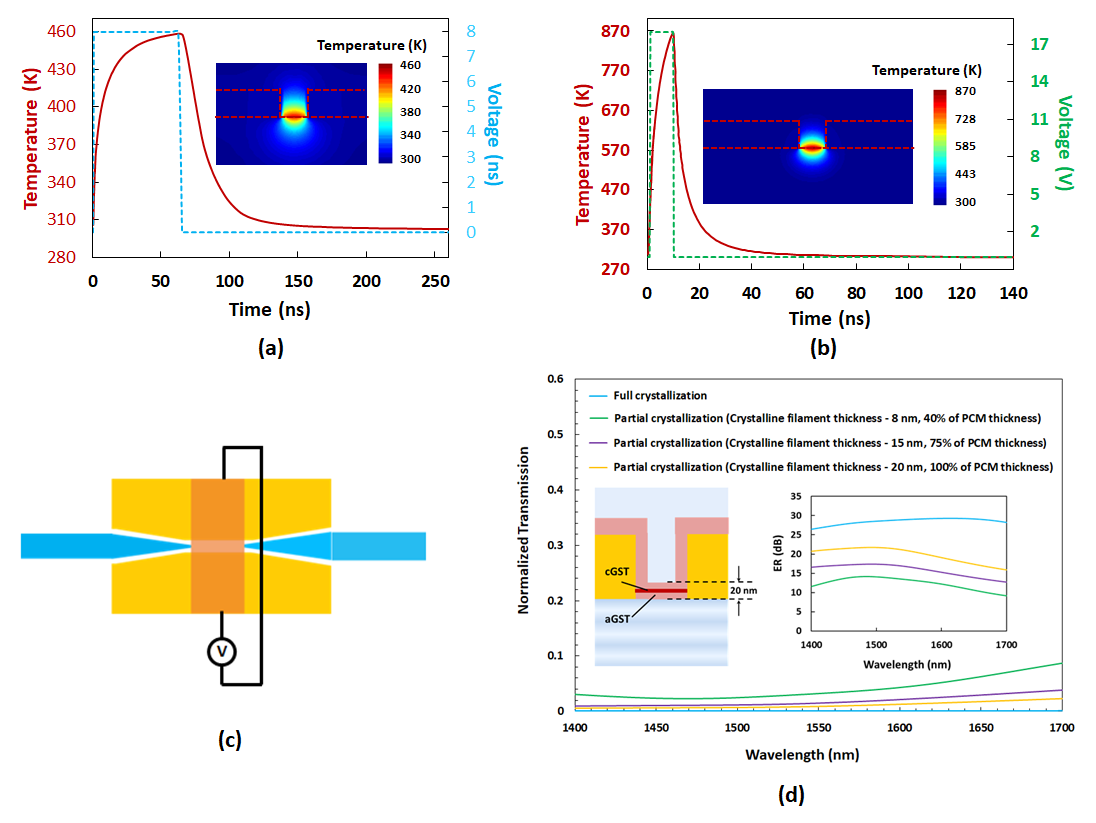


**Figure S3.** The transient temperature response due to the electrical threshold switching by an applied pulse of: (a) 8 V for 65 ns in the crystallization process and (b) 18 V for 10 ns in the amorphization process. The insets illustrate the spatial distributions of the temperature profile in the 2D cross-sections of the plasmonic slot waveguide. (c) A schematic showing how the hybrid plasmonic switch is connected with a voltage source using a high-speed transmission line (such as a copper interconnect). (d) Transmission spectra for both the cases i.e., partial crystallization and full crystallization of the GST film. The insets illustrate the ER spectrum and the cross sectional view of the slot waveguide with crystalline GST filament.

amorphous phase. The effect of the crystallization of the GST film ⎯ when only the GST film on the bottom of the slot waveguide is crystallized (partial crystallization) as compared to that when the entire GST film layer is crystallized (full crystallization) ⎯ on the transmission spectra is shown in Fig. S3(d). Many crystalline filaments with different volume are formed between the electrodes at the bottom portion of the slot waveguide. To consider this effect, we have taken a uniform thin film of a crystalline GST within the amorphous GST film. The effect of the crystallization of different fractions of the GST film volume (40%, 75% and 100% of the GST thickness) on the transmission in OFF state, and the corresponding ER, is shown in Fig. S3(d). It shows a small amount of increase in the transmission and lower ER in the case of partial crystallization compared to full crystallization of the GST film. But the ER is significantly high enough from the previously reported results^16^. This behavior is expected, as the plasmonic slot waveguide mode is mainly confined in the bottom portion of the plasmonic slot waveguide in the crystalline phase (see Fig. 1(c) of the main paper). Therefore, the light-matter interaction in the bottom portion is significantly high compared to the other portions. Fig. S4 shows two other possible configurations based on electrical conduction heating mechanism. In the first configuration, the same metal waveguide can be used as a nano-heater, which leads to a compact device size as no separate heater is required (See Fig. S4 (a), (b)). It also supplies a significant heating through the GST metal contacts for full volume crystallization. On other hand, undoped single layer graphene can be use as a micro-heater to thermally change the phase of the GST (See Fig. S4 (c), (d)). We can achieve the required temperature for crystallization and amorphization by applying a voltage across the graphene layer. This is also an effective way for electrical conduction heating in PCM applications, as the graphene has high electrical and thermal conductivity.


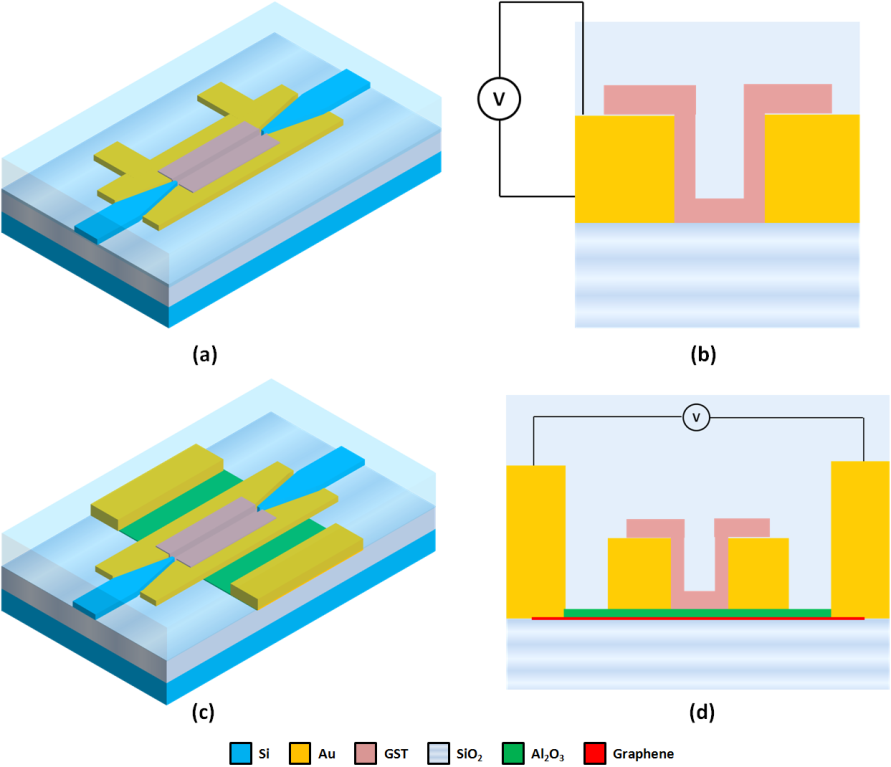


**Figure S4.** (a) Schematic of the proposed broadband non-volatile hybrid electro-optic plasmonic switch based on thermal conduction heating via metal micro-heater. (b) The cross-sectional view of the plasmonic slot waveguide with the metal micro-heater connected to a voltage source. (c) Schematic of the proposed broadband non-volatile hybrid electro-optic plasmonic switch based on thermal conduction heating via graphene micro-heater. (d) The cross-sectional view of the plasmonic slot waveguide with the graphene micro-heater connected to a voltage source.

Table S2. Thermoelectric properties of different materials used in the simulations

| Material | Heat capacity  C (J/Kg K) | Thermal Conductivity K (W/mK) | Electrical Conductivity σ (S/m) | Density  ρ (Kg/m^3^) |
| --- | --- | --- | --- | --- |
| SiO_2_ | 709 | 1.38 | 1×10^-11^ | 2203 |
| Si | 711 | 148 | 102 | 2330 |
| Au | 129 | 316 | 4.005×10^7^ | 19300 |
| aGST | 213 ^[17]^ | 0.19 ^[7]^ | σ (T) ^[18]^ | 5870 ^[19]^ |
| cGST | 199 ^[17]^ | 0.57 ^[7]^ | σ (T)  ^[18]^ | 6300 ^[19]^ |

**7. Performance of plasmonic logic gates and circuits**

The total time delay for all the proposed architectures is primarily dependent on the time delay involved in the phase transition (summation of voltage pulse width and the thermal decay time) of a single plasmonic switch (i.e., for the amorphization and/or crystallization process), as the input signals can be applied simultaneously, and there is the negligible propagation delay in optical interconnects compared to the phase transition time. The phase transition time for the crystallization process is higher than the amorphization process. Therefore, the switching speed of the plasmonic logic gates is limited by the applied voltage pulse width for crystallization and the thermal decay time or the relaxation time. The decay time is defined as the time for the decay from peak temperature to (1/e) of the peak temperature. The required voltage pulse width and decay time for crystallization are 65 ns and 25 ns, respectively. Therefore, the transition speed for all the logic gates is ~ 90 ns. Table S3 shows a comparison of the performance (in terms of energy consumption, area, speed, and extinction ratio) of our proposed plasmonic logic gates with that of the different previously reported optical logic gates.

**Table S3. Comparison of our proposed plasmonic logic gates with previously reported optical logic gates**

| **Device Structure** | **Logic operations** | **Energy consumption** | **Active material area** | **Speed** | **Extinction ratio (ER)** | **Volatility** |
| --- | --- | --- | --- | --- | --- | --- |
| Carrier-depletion based micro-ring resonators [20] | AND, OR, XOR, XNOR | 1200 fJ | 8000 µm^2^ | 3 Gbps | 14 dB | Volatile |
| Two-photon absorption in silicon microring waveguides [21] | AND, NAND | 3200 fJ | 90 µm^2^ | 310 Mbps | 10 dB | Volatile |
| Multi-operand carrier dispersion effect in microring resonator [22] | NAND, OR, XOR | 20 fJ | 400 µm^2^ | 50 Gbps | 10 dB | Volatile |
| Thermo-optic effect in microring resonator [23] | XOR, XNOR | - | 1.5×0.6 mm^2^ | 20 kbps | 15 dBm | Volatile |
| Device-Level Logic operations Using Phase-Change Materials [24] | OR, NAND | 5 mW | 2.6 µm^2^ | 90 ns | 5% change in transmission b/w two logic states | Non-Volatile |
| Plasmonic switch (This work) | AND, OR, NAND, NOR, XOR, XNOR, NOT, D-Latch, SR-Latch, JK-Latch, T-Latch, Half-Adder | 716 pJ | 0.09 µm^2^ – 0.54 µm^2^ | 90 ns | 21 dB – 35 dB | Non-Volatile |

# 8. Sequential non-volatile asynchronous EO logic (Latch) circuits

D-latch or transparent latch is a one-input SR latch and it removes the possibility of invalid input states of the SR latch. To implement the D latch circuit, we have used the non-inverting plasmonic switch in the upper arm (instead of an inverting EO plasmonic switch in the SR latch), and both the inputs are connected to the D-latch. When the input of the latch is logic ‘0’, the outputs are $Q_{n+1}$= 0 and $\bar{Q}_{n+1}$ = 1 (Reset state). When the input of the latch is logic ‘1’, the outputs are $Q_{n+1}$ = 1 and $\bar{Q}_{n+1}$ = 0 (Set state). The architecture, truth table and graphical symbol are shown in Fig 7(b) of the main paper. The designs of the photodetector and the conditional electronic circuit are beyond the scope of paper.

To design the JK latch circuit, we have used a total of six plasmonic switches of inverting logic. The JK flipflop is based on the NOR logic. The output of a JK flipflop can be expressed as, $Q_{n+1}=J{\cdot\bar{Q}}_{n}+{\bar{K}.Q}_{n}$ and $\bar{Q}_{n+1}=K.Q_{n}+\bar{J.}\bar{Q}_{n}$. The electro-optic configuration of the JK latch circuit is shown in Fig. 8(a) of the main paper. When both inputs (J and K) are in logic ‘0’, optical mode passes through the first inverting EO switch of the NAND gate of both arms and then it passes through the second EO switch depending on the previous outputs. If $Q_{n}$ is logic ‘0’ (i.e., $\bar{Q}_{n}$ = 1), optical modes can propagate through the NAND gate and the output of the NAND gate of both arms is logic ‘1’ irrespective of the previous output. The output of the NAND gate is multiplied with the $\bar{\bar{Q}_{n}}$ using another inverting EO switch in the upper arm and the output of the switch $Q_{n+1}$ is logic ‘0’. Due to the low intensity, the electrical signal in the photodetector (PD1) output is also low. Thus, the conditional electronic circuit generates a voltage pulse for crystallization. This changes the phase of the GST in the EO switch in the lower arm. As a result, the optical mode passes through the EO switch and the output of the NAND gate is logic ‘1’. Thereafter, the optical output is multiplied with the $\bar{Q_{n}}$ using the inverting EO switch and the final output $\bar{Q}_{n+1}$ is ‘1’. Thus, the state of the JK latch is the same as the previous state, which is called the ‘No change state’. When the inputs are J = 0, K = 1, and the previous outputs are $Q_{n}$ = 1, $\bar{Q}_{n}$ = 0, the optical mode can’t propagate through the NAND gate in the upper arm. Thus, the output of the NOR gate $Q_{n+1}$ is logic ‘0’. The photodetector output (PD1) is low and the conditional circuit generates the electric pulse for crystallization. As a result, optical mode propagates through the NAND gate in the lower arm. Thereafter, the optical output of the NAND gate is multiplied with the $\bar{Q_{n}}$ using the inverting EO switch and the final output $\bar{Q}_{n+1}$ is logic ‘1’. This state is called the ‘Reset’ state. Similarly, for inputs J = 1 and K = 0, the output $Q_{n+1}$= 1 and $\bar{Q}_{n+1}$ = 0 (Set state). When both the inputs are logic ‘1’ and the previous output states are $Q_{n}$ = 1, $\bar{Q}_{n}$ = 0, the optical mode can’t mode propagate through the NAND gate in the upper arm. Thus, the output of the NOR gate $Q_{n+1}$ is logic ‘0’. The photodetector output (PD1) is low and the conditional circuit generates the electric pulse for crystallization. As a result, the optical mode can propagate through the NAND gate and the output of the NOR gate in the lower arm $\bar{Q}_{n+1}$ is logic ‘1’. In the next state, the photodetector output (PD1) is low and the conditional circuit generates the electric pulse for crystallization. Thus, the output of the NOR gate in the upper arm $Q_{n+2}$ is logic ‘1’. Similarly, the output in the lower arm $\bar{Q}_{n+2}$ is logic ‘0’. Thus, the output is toggled at each state, which is called as ‘Toggle state’. The corresponding truth table and the electrical schematic are also shown in Fig. 8(a) of the main paper. The calculated extinction ratio for this EO JK latch is 28.36 dB. Similarly, to implement the T latch circuit, both inputs of the JK latch are connected to the input signal of the T latch. When the input of the T latch is logic ‘0’, the outputs are the same as the previous state (No change state). When the input of the latch is logic ‘1’, the outputs are toggle (Toggle state). The architecture, the truth table, and the graphical symbol are shown in Fig 8(b) of the main paper.

# References

1. Yu, Z., Zheng, J., Xu, P., Zhang, W. & Wu, Y. Ultracompact electro-optical modulator-based Ge_2_Sb_2_Te_5_ on silicon. IEEE Photonics Technology Letters 30, 250-253 (2017).
2. Liang, H. et al. Simulations of Silicon-on-Insulator Channel-Waveguide Electrooptical 2× 2 Switches and 1× 1 Modulators Using a Ge_2_Sb_2_Te_5_ Self-Holding Layer. Journal of Lightwave Technology 33, 1805-1813 (2015).
3. Raoux, S., Xiong, F., Wuttig, M. and Pop, E. Phase change materials and phase change memory. *MRS bulletin* **39,** 703-710 (2014).
4. Ríos, C. et. al. Integrated all-photonic non-volatile multi-level memory. *Nature photonics* **9,** 725-732 (2015).
5. Raoux, S. et. al. Phase-change random access memory: A scalable technology. *IBM Journal of Research and Development* **52,** 465-479 (2008).
6. Burr, G.W. et al. Phase change memory technology. *Journal of Vacuum Science & Technology B, Nanotechnology and Microelectronics: Materials, Processing, Measurement, and Phenomena* *28*, 223-262 (2010).
7. Ríos, C. et al. Integrated all-photonic non-volatile multi-level memory. *Nature Photonics* **9**, 725-732 (2015).
8. Markov, P., Appavoo, K., Haglund, R. F. and Weiss, S. M. Hybrid Si-VO_2_-Au optical modulator based on near-field plasmonic coupling. *Optics express* **23,** 6878-6887 (2015).
9. Kato, K., Kuwahara, M., Kawashima, H., Tsuruoka, T. and Tsuda, H. Current-driven phase-change optical gate switch using indium–tin-oxide heater. *Applied Physics Express* **10,** 072201 (2017).
10. Rudé, M. et. al. Optical switching at 1.55 μm in silicon racetrack resonators using phase change materials. *Applied Physics Letters* **103,** 141119 (2013).
11. Faneca, J., Bucio, T.D., Gardes, F.Y. and Baldycheva, A. O-band N-rich silicon nitride MZI based on GST. *Applied Physics Letters* **116**, 093502 (2020).
12. Zhang, H. et al. All-optical non-volatile tuning of an AMZI-coupled ring resonator with GST phase-change material. *Optics letters* **43**, 5539-5542 (2018).
13. Faneca, J., Bucio, T. D., Gardes, F. Y., and Baldycheva, A. O-band n-rich silicon nitride MZI based on GST. *Applied Physics Letters* **116**, 093502 (2020).
14. Zhang, H. et al. Ultracompact Si-GST hybrid waveguides for non-volatile light wave manipulation. *IEEE Photonics Journal* **10**, 1-10 (2017).
15. Pirovano, A. et al. Scaling analysis of phase-change memory technology. *In IEEE International Electron Devices Meeting*, 29-6 (2003).
16. Farmakidis, N. et al. Plasmonic nanogap enhanced phase-change devices with dual electrical-optical functionality. *Science advances* 5(11), eaaw2687 (2019).
17. Lyeo, H. K. et al. Thermal conductivity of phase-change material Ge_2_Sb_2_Te_5_. *Applied Physics Letters* **89**, 151904 (2006).
18. Xiong, F., Liao, A. D., Estrada, D., & Pop, E. Low-power switching of phase-change materials with carbon nanotube electrodes. *Science* **332**, 568-570 (2011).
19. Njoroge, W. K., Wöltgens, H. W. & Wuttig, M. Density changes upon crystallization of Ge_2_Sb_2.04_Te_4.74_ films. *Journal of Vacuum Science & Technology A: Vacuum, Surfaces, and Films* **20**, 230-233 (2002).
20. Qiu, C., Gao, W., Soref, R., Robinson, J.T. and Xu, Q. Reconfigurable electro-optical directed-logic circuit using carrier-depletion micro-ring resonators. *Optics letters* **39,** 6767-6770 (2014).
21. Xu, Q. and Lipson, M. All-optical logic based on silicon micro-ring resonators. *Optics express* **15,** 924-929 (2007).
22. Ying, Z. et. al. Integrated multi-operand electro-optic logic gates for optical computing. *Applied Physics Letters* **115**, 171104 (2019).
23. Zhang, L. et. al. Demonstration of directed XOR/XNOR logic gates using two cascaded microring resonators. *Optics letters* **35**, 1620-1622 (2010).
24. Cheng, Z. et. al. Device-level photonic memories and logic applications using phase-change materials. *Advanced Materials* **30**, 1802435 (2018).
